# Socio-Demographic, Lifestyle, and Cardiometabolic Characteristics Associated with Low-Grade Systemic Inflammation in Russian Adult Population

**DOI:** 10.3390/biom13050835

**Published:** 2023-05-14

**Authors:** Olga Mirolyubova, Kamila Kholmatova, Anna Postoeva, Galina Kostrova, Sofia Malyutina, Alexander V. Kudryavtsev

**Affiliations:** 1 Department of Faculty Therapy, Northern State Medical University, Arkhangelsk 163069, Russia; 2 International Research Competence Centre, Northern State Medical University, Arkhangelsk 163069, Russia; 3 Department of Hospital Therapy and Endocrinology, Northern State Medical University, Arkhangelsk 163069, Russia; 4 Department of Community Medicine, UiT the Arctic University of Norway, N-9037 Tromsø, Norway; 5 Department of Normal Physiology, Northern State Medical University, Arkhangelsk 163069, Russia; 6 Research Institute of Internal and Preventive Medicine, Branch of Institute of Cytology and Genetics, Siberian Branch of the Russian Academy of Sciences, Novosibirsk 630008, Russia; 7 Department of Therapy, Hematology and Transfusiology, Novosibirsk State Medical University, Novosibirsk 630091, Russia

**Keywords:** low-grade systemic inflammation, high-sensitivity C-reactive protein, socio-demographic factors, lifestyle, abdominal obesity, dyslipidemia, hypertension, smoking, Russia

## Abstract

Mortality from cardiovascular diseases (CVDs) is higher in Russia compared to other European countries. High-sensitivity C-reactive protein (hs-CRP) is a biomarker of inflammation, and its elevated levels indicate increased CVD risks. We aim to describe the prevalence of low-grade systemic inflammation (LGSI) and the associated factors in a Russian population. The Know Your Heart cross-sectional study was conducted in Arkhangelsk, Russia in 2015–2017 with a population sample aged 35–69 years (*n* = 2380). LGSI was defined as hs-CRP ≥ 2 and <10 mg/L, and its associations with socio-demographic, lifestyle, and cardiometabolic characteristics were analyzed. The prevalence of LGSI (age-standardized to European Standard Population 2013) was 34.1% (33.5% in men and 36.1% in women). In the total sample, the increased odds ratios (ORs) of LGSI were associated with abdominal obesity (2.1), smoking (1.9), dyslipidemia (1.5), pulmonary diseases (1.4), and hypertension (1.3); the decreased ORs were in women (0.6) and in married participants (0.6). In men, the ORs were higher with abdominal obesity (2.1), smoking (2.0), CVDs (1.5), and hazardous drinking (1.5); in women—with abdominal obesity (4.4) and pulmonary diseases (1.5). In conclusion, one-third of the adult population in Arkhangelsk had LGSI. Abdominal obesity was the strongest LGSI correlate in both sexes, while the profiles of other associated factors were different between men and women.

## 1. Introduction

C-reactive protein (CRP) is a biomarker of inflammation [1,2,3]. High-sensitivity CRP (hs-CRP) tests are commonly used to assess CRP levels. High levels (≥10 mg/L) of hs-CRP indicate acute inflammatory processes (infections, injuries, surgery, etc.). The level between 2 and 10 mg/L indicate a low-grade (subclinical) systemic inflammation (LGSI), which has associations with chronic non-infectious diseases, including cancer, diabetes, and cardiovascular diseases (CVD) [4,5,6,7]. The nature of the relations between inflammation and chronic conditions is not entirely clear [1,4,5].

Mortality from CVDs in Russia exceeds that in the majority of European countries, especially among middle-aged men [8]. The Know Your Heart Study (2015–2018) aimed to investigate the causes of the high premature CVD in the Russian population [9]. Comparisons of the KYH data with data from Norway (the seventh Tromsø study) have found similar levels of total cholesterol (TC), low-density lipoprotein cholesterol (LDL-C), and high-density lipoprotein cholesterol (HDL-C) [10]. This was an unexpected finding, given dyslipidemia is one of the main CVD risk factors, and the CVD mortality in Russia exceeds that in Norway by the factor of four [8]. In parallel, the comparisons showed higher levels of hs-CRP in the Russian study, along with elevated population levels of biomarkers of heart damage (cardiac troponin T and N-terminal pro-b-type natriuretic peptide) [10]. The finding of different hs-CRP levels but almost the same cholesterol fractions also challenged the existing knowledge of metabolic syndrome, which is a known associate of both dyslipidemia and chronic inflammation [11,12,13]. The explanations might be in comparisons of population levels of remnant cholesterol and oxidized LDL-C, which were not performed. For instance, remnant cholesterol was shown causally associated with LGSI, atherogenicity, and ischemic heart disease [14,15]. Oxidized LDL-C was shown as leading to atherosclerotic damages in the vascular wall and apoptosis induction mediated by Fas/Fas ligand death pathway [16]. In addition, the findings in the Russian–Norwegian comparison may be due to a shared genetic predisposition to inflammation and selected cardiometabolic phenotypes [17,18,19], but this hypothesis also remains untested.

The elevated levels of inflammation in Russia may also be due to specific socio-economic and lifestyle characteristics [20,21,22,23,24]. For instance, higher CRP levels can be associated with lower education levels and a poor financial situation [23,24]. The level of alcohol intake may be important as it demonstrated J- and U-shaped relationships with CRP, and heavy drinking was shown to cause specific damages to body tissues, cardiac remodeling, and alcohol-related cardiomyopathy [20,21,25,26]. The correction of lifestyle factors (physical activity, diet, alcohol consumption, and smoking) was demonstrated to reduce inflammation levels [4,22,27,28,29,30].

Based on the weaknesses of the existing explanations of the earlier observed elevated levels of inflammation in Russia’s population, the aim of our study is to describe the distribution of LGSI in the adult population of Arkhangelsk, Russia and investigate its socio-demographic, lifestyle, and cardiometabolic correlates.

## 2. Materials and Methods

### 2.1. Study Population

We used data from the Know Your Heart (KYH) cross-sectional study of cardiovascular diseases, which was conducted in Arkhangelsk and Novosibirsk in 2015–2018 as a part of the international project on cardiovascular disease in Russia (https://knowyourheart.science/; accessed on 8 May 2023). In Arkhangelsk, the KYH study population comprised men and women aged 35–69 years (*n* = 2380), randomly sampled from the general population, and representing 1.5% of the total residents in the age span. The participants were sampled using the depersonalized database of the Arkhangelsk territorial health insurance fund, containing information about all residents with mandatory health insurance. The dataset accessed for the sampling purposes contained anonymous addresses of the insured residents aged 35–69 years, supplemented by age and sex variables. Trained interviewers visited randomly selected addresses. When a contact at an address was made, a household member of the defined age (±2 years) and sex was invited to take part in the study. Among the invited, 68% agreed and had an interview at home. The interview participates were offered a health examination at the polyclinic of the Northern State Medical University, and 96% underwent the examination. Details of the KYH study rationale, design, sampling, recruitment, and measurement procedures were published earlier [9].

### 2.2. Data Collection

Trained interviewers conducted at-home (baseline) interviews and collected data on participants’ demographic, socio-economic, and lifestyle characteristics, self-reported diseases, and use of healthcare services. Trained physicians and nurses conducted medical examinations at the polyclinic, including a medical interview (history and symptoms of diseases, medication use), taking blood samples, and a series of instrumental and functional measurements of cardiovascular health and related parameters. The participants were asked to fast for four hours prior to the examination; therefore, blood samples were taken in four or more hours after the last meal. The samples were centrifuged with the serum frozen at −80 °C. When the fieldwork was finished, the samples were analyzed in a single batch.

### 2.3. Low-Grade Systemic Inflammation

LGSI was defined based on the serum level of hs-CRP, measured using immuno-turbidimetric test (AU 680 Chemistry System; Beckman Coulter, Tokyo, Japan). The participants with missing hs-CRP data (*n* = 10) were excluded from all analyses. The participants with hs-CRP ≥ 2 mg/L and <10 mg/L were categorized as LGSI-positives (LGSI+), and those with hs-CRP < 2 mg/L were treated as LGSI-negatives (LGSI-). The participants with hs-CRP ≥ 10 mg/L, which was considered an indication of an acute inflammatory process, were included when assessing hs-CRP levels and the LGSI prevalence in the studied population, but were excluded from analyses of LGSI associations with socio-economic, lifestyle, and cardiometabolic characteristics.

### 2.4. Socio-Demographic Characteristics

To describe the distribution of hs-CRP levels and the LGSI prevalence in the population and investigate their socio-demographic correlates, we used the following variables: age (years), sex (man or woman), marital status (in registered marriage, not in registered marriage), higher education (yes or no), poor financial situation (self-reported difficulties to buy food or clothes, yes or no). In addition, occupation was analyzed as having regular paid work (yes or no) and as four occupation categories according to the International Standard Classification of Occupation (ISCO) [31]: high-skilled white-collars (ISCO 1–3: legislators, senior officials and managers, professionals, technicians, and associate professionals), low-skilled white-collars (ISCO 4–5: clerical support workers, service, and sales workers), high-skilled blue-collars (ISCO 6–7: skilled agricultural, forestry and fishery workers, and craft and related trades workers), and low-skilled blue-collars (ISCO 8–9: plant and machine operators or assemblers, elementary occupations).

### 2.5. Housing Characteristics

To assess LGSI associations with living conditions, we used data on participants’ housing characteristics (shared flat/house or hostel, absence of hot water amenities, absence of central heating; yes or no for each of the three), household size (number of people living together), and dwelling size (size of apartment or house) per one household member (m^2^).

### 2.6. Lifestyle Characteristics

The lifestyle characteristics considered were smoking, hazardous alcohol drinking, physical activity, and diet. Smoking was defined as self-reported current daily smoking (yes or no). Hazardous drinking was defined as a score ≥8 on the Alcohol Use Disorders Identification Test (AUDIT) [32]. Physical activity was measured using the EPIC physical activity questionnaire and categorized into four levels (inactive, moderately inactive, moderately active, and active) by applying the ‘‘total physical activity index’’, which was developed based on quartiles of metabolic equivalent of task (MET) values estimated for occupational and recreational activities of varying intensity and duration [33]. In this study, a participant was defined as physically inactive if belonging to “inactive” or “moderately inactive” category. Diet was assessed using the dietary quality score questionnaire [34], which takes into account the frequency of fish, fruits, and vegetables intake and type of fats used, giving a total score of 0–8 points where 0–2, 3–5, and 6–8 points are labelled as unhealthy, average, and healthy diet, respectively. The latter two categories were merged, and the as the variable used was “unhealthy diet” (yes or no).

### 2.7. Cardiometabolic Characteristics

Cardiometabolic correlates of LGSI and hs-CRP levels were analyzed at several levels, starting with directly measurable blood biomarkers and instrumentally measured body parameters, proceeding to composite conditions (dyslipidemia, hypertension, diabetes, metabolic syndrome), and ending with diagnosed diseases.

Levels of TC (mmol/L), HDL-C (mmol/L), LDL-C (mmol/L), and triglycerides (TG) (mmol/L) were assessed in the blood serum using enzymatic color tests; apolipoprotein A-I (Apo A1) (g/L), apolipoprotein B (Apo B) (g/L), and glycated hemoglobin (HbA1c) (%)—using immuno-turbidimetric tests, lipoprotein(a) (Lp(a)) (mg/dl)—using a particle-enhanced immuno-turbidimetric test (AU 680; Chemistry System Beckman Coulter) [9]. Apo B/Apo A-1 ratio was calculated as Apo B divided by Apo A-1. The level of non-HDL cholesterol (mmol/L) was calculated by subtracting HDL-C from TC. The level of remnant cholesterol (mmol/L) was calculated in two steps: (a) estimated LDL-C (LDL-C_est_) was calculated using Friedewald equation (LDL-C_est_ = TC − (HDL-C + TG/2.2)) [35], and then (b) remnant cholesterol was calculated by subtracting HDL-C and LDL-C_est_ from TC. If TG levels were >4 mmol/l, directly measured LDL-C values were used in the formulae.

Systolic and diastolic blood pressure (SBP and DBP) (mm Hg) were measured on the brachial artery using an OMRON 705 IT automatic blood pressure monitor (OMRON Healthcare). The measurements were performed three times at two-minute intervals. SBP and DBP were analyzed as means of the second and third measurements.

Height (cm) was measured using a Seca^®^ 217 stadiometer (Seca Ltd., Hamburg, Germany). Weight (kg) was measured using TANITA BC 418 body composition analyzer (Tanita Corp., Tokyo, Japan). Waist circumference (WC) (cm) and hip circumference (HC) (cm) were measured using Seca^®^ 201 measuring tape (Seca Limited). WC was measured at the narrowest part of the trunk. Height, WC, and HC were measured twice, and the averages of the two measurements were used in the analysis. Body mass index (BMI) was calculated as weight in kilograms divided by squared height in meters. Waist-to-hips ratio (WHR) was calculated as WC divided by HC. Abdominal obesity was defined as WC > 94 cm for men, or > 80 cm for women.

Medication data were collected by asking the participants about their currently used medicines. The commercial name, dose, indication, and frequency were recorded for up to seven medications. The recorded medications’ names were coded using the international WHO anatomical therapeutic chemical (ATC) classification system version 2016 [36,37]. Lipid-lowering medication (statins) was defined as any medication within the ATC class C10. Antihypertensive medication was defined as any medication coded as ATC classes C02, C03, C07, C08, or C09; antidiabetic medication was any medication coded as ATC class A10. Anti-inflammatory medication was defined as any medication within the ATC classes A07E, G02CC, M01A, M01B, M02AA, S01B, S01C, or H02.

Dyslipidemia was defined as TC ≥ 5.2 mmol/L and/or TG > 1.7 mmol/L and/or LDL-C > 3.0 and/or HDL-C < 1.0 mmol/L for men and/or HDL-C < 1.2 mmol/L for women and/or intake of lipid lowering medication. Hypertension was defined as SBP > 140 mm Hg and/or DBP > 90 mm Hg and/or self-reported intake of antihypertensives. Diabetes was defined as HbA1c ≥ 6.5% and/or self-reported intake of antidiabetics and/or self-report of having been diagnosed with diabetes, followed by the statement of the diabetes type and the treatment prescribed (insulin, drugs, or diet).

According to the AHA/NHBLI (2009) criteria, metabolic syndrome (MS) was defined [38] as having any three of the following five criteria: (1) WC ≥ 94 cm in men and ≥ 80 cm in women; (2) TG > 1.7 mmol/L and/or lipid-lowering medication; (3) HDL-C < 1.0 mmol/L for men and <1.3 mmol/L for women; (4) SBP > 130 mm Hg and/or DBP > 85 mm Hg and/or antihypertensive medication; (5) HbA1c ≥ 5.7% and/or antidiabetic medication.

### 2.8. Self-Reported Diseases

Data on the history of diseases were collected by asking the participants about having ever been diagnosed with a CVD (angina pectoris, myocardial infarction or heart attack, stroke, heart failure, or atrial fibrillation), a pulmonary disease (bronchial asthma, chronic obstructive pulmonary disease, or chronic bronchitis), a joint disease (rheumatoid arthritis, osteoarthritis, or osteoarthritis), diabetes mellitus, cancer, a kidney disease, or a liver disease. In addition, we used answers to questions about a prior heart surgery (percutaneous coronary intervention or coronary bypass surgery) and clarifying questions to the participants who self-reported diabetes (diabetes type, control with insulin, drugs, or diet). The listed diseases were considered present if the corresponding diagnoses were self-reported.

### 2.9. Mental Health

Depression was measured using the patient health questionnaire-9 (PHQ-9), and the cut point of ≥5 was used to define any depression (mild, moderate, major, or major severe) [39]. The anxiety level was assessed using the general anxiety disorder-7 (GAD-7) questionnaire, and scores ≥ 5 were used to identify those with any anxiety (mild, moderate, or severe) [40].

### 2.10. Exclusions from the Study

Analyses of hs-CRP population levels and the LGSI prevalence were performed on the sample of 2370 KYH participants, after excluding 10 KYH participants with missing hs-CRP data. Further analyses of LGSI correlates were performed on the sample of 2054 participants, after exclusions of 158 participants with hs-CRP concentration > 10 mg/L, 61 participants who self-reported taking anti-inflammatory medication, and 97 participants with missing data on any of the analyzed variables.

### 2.11. Statistical Analysis

Absolute numbers (Abs) and proportions (%) were used to present categorical variables. Proportions representing prevalence estimates were shown with 95% confidence intervals (CIs). Age-standardized proportions were calculated based on the European Standard Population 2013 in the age span of 35–69 years, with 5-year intervals. Mean values (Ms) with standard deviations (SDs) and/or medians (Mes) with quartiles (Q1; Q3) were used to present continuous and count variables. In selected analyses, skewed continuous variables were included in ln-transformed form and presented as geometric means.

Analyses of LGSI associations with socio-demographic, lifestyle, and cardiometabolic characteristics were performed stratified by sex and on the pooled sample with significant interactions between sex and other socio-demographic, lifestyle, and cardiometabolic covariates considered. For each covariate, interaction with sex was assessed by comparing regression models with and without the interaction term using the likelihood ratio test.

Comparisons of the LGSI-positive and LGSI-negative participants on categorical variables were made using Pearson’s chi-squared test and Cochran–Armitage test for trend where relevant. Differences between the two groups on normally distributed continuous variables were assessed using a two-sample t-test on continuous variables with skewed distributions and on count variables—using two-sample Wilcoxon rank-sum test and Jonckheere–Terpstra test for trends where relevant. Parameters that differed between the LGSI-positive and LGSI-negative participants in men and/or in women were entered into further multivariable analyses of the associations with LGSI.

The LGSI associations with socio-demographic, lifestyle, and cardiometabolic characteristics were assessed using multivariable logistics regressions with stepwise entry of covariates. First, LGSI associations with all studied parameters, except age, were adjusted for age (Model 1). Second, socio-demographic and lifestyle variables (age, sex, higher education, being married, being in regular paid work, occupational category, smoking, and hazardous drinking) were mutually adjusted, with the addition of significant interaction terms sex by being married and sex by smoking (Model 2). Next, we added cardiometabolic conditions (dyslipidemia, hypertension, diabetes, abdominal obesity) and interaction term sex by abdominal obesity (Model 3). Finally, we added self-reported diseases (cardiovascular, pulmonary, and joint diseases) (Model 4). Based on the identified interactions of several variables with sex, the same analyses were repeated for men and women separately. Additionally, analyses of the earlier identified independent correlates of LGSI were repeated with MS variable entered instead of a set of previously used cardiometabolic conditions (separate MS components). This was performed on the total sample using multivariable logistic regression with all variables mutually adjusted and added interaction terms sex by marital status and sex by smoking. To address the identified interactions, these analyses were also performed stratified by sex. Results of these analyses were presented as adjusted odds ratios (ORs) with 95% CIs.

The strength of the associations between the hs-CRP level and cardiometabolic biomarkers, which are conventionally assessed at a screening or a doctor visit (lipid levels, blood pressure, blood glucose, adiposity-related anthropometric parameters, and use of related medications), was assessed using multivariable linear regressions with the ln-transformed hs-CRP level as the dependent variable and with adjustment for age and sex. Results of these analyses are presented as standardized β coefficients, which allow comparing the strengths of the discovered associations.

All analyses were performed using STATA V.17 (StataCorp, College Station, TX, USA).

## 3. Results

**Figure 1:**
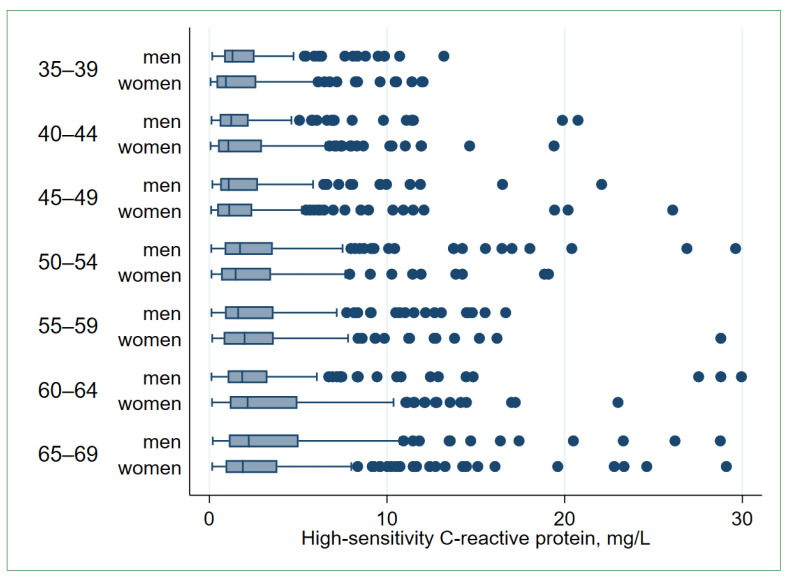
High-sensitivity C-reactive protein (hs-CRP) levels in the study population by age and sex (*n* = 2343). The graph is built after excluding 27 observations with hs-CRP > 30 mg/L. Boxes indicate inter-quartile ranges (IQRs) divided by the median line, whiskers—adjacent values within 1.5 IQR of the nearer quartile, dots—outliers. Jonckheere–Terpstra test for trend: *p*_men_ < 0.001, *p*_women_ < 0.001.

The studied population (*n* = 2370) comprised 987 men (41.6%) and 1383 women (58.4%). The mean age was 53.8 years, equal for men and women. The median hs-CRP levels were 1.59 (Q1 = 0.71; Q3 = 3.54) mg/L in the total (pooled) study population, 1.65 (Q1 = 0.79; Q3 = 3.56) mg/L in men, and 1.50 (Q1 = 0.64; Q3 = 3.54) mg/L in women. The median hs-CRP levels increased with age in both sexes (Figure 1, Table S1). The geometric mean for the hs-CRP level was 1.62 (95% CI: 1.55; 1.70) mg/L in the total study population, 1.74 (95% CI: 1.62; 1.87) mg/L in men, and 1.54 (95% CI: 1.45; 1.64) mg/L in women (*p* = 0.012).

**Figure 2:**
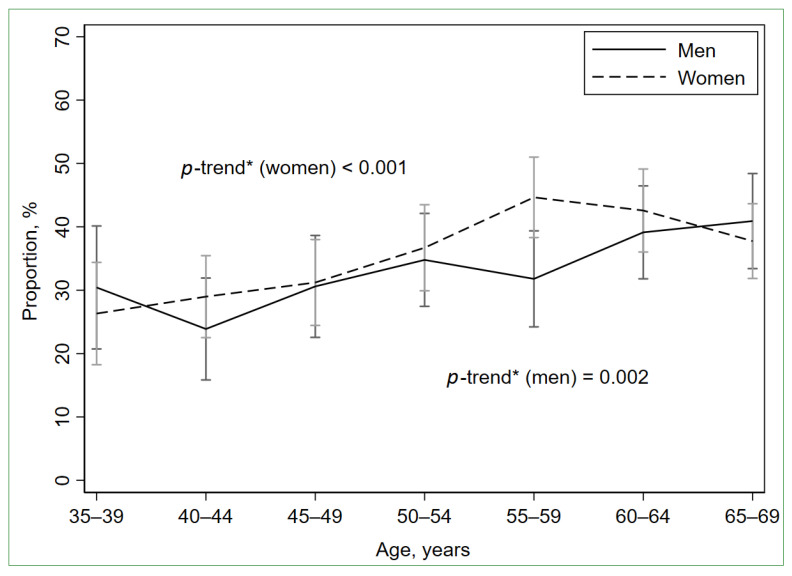
Prevalence of low-grade systemic inflammation (hs-CRP ≥ 2 mg/L and <10 mg/L) by age and sex. Error bars indicate 95% confidence intervals. * Cochran–Armitage test for trend.

The participants with LGSI (*n* = 830) constituted 35.0% (95% CI: 33.1%; 37.0%) of the total study population, 33.5% (95% CI: 30.7%; 36.5%) of men (*n* = 331), and 36.1 (95% CI: 33.6%; 38.7%) of women (*n* = 499) (*p* = 0.200). Age-standardized to the European Standard Population 2013, the proportion of the participants with LGSI was 34.1% (95% CI: 32.2%; 36.1%) for the total study population, 32.7% (95% CI: 29.8%; 35.7%) for men, and 35.1 (95% CI: 32.6%; 37.7%) for women. The proportions of LGSI-positive participants were growing with age in both men and women (Figure 2, Table S2).

### 3.1. Comparisons of the LGSI-Positive and LGSI-Negative Participants

Among both men and women, LGSI-positive participants were on average older and had lower proportions of those in regular paid work (Table 1), compared LGSI-negatives. In men, the LGSI-positives had a lower proportion of married participants, but higher proportions of smokers and hazardous drinkers. In women, the LGSI-positives had lower proportions of those highly educated and high-skilled white-collars. The LGSI-positives did not differ from the LGSI-negatives in financial situation, housing characteristics, dietary quality, and physical activity.

Dyslipidemia, hypertension, and abdominal obesity were more frequently observed in the LGSI-positive men and women, compared to the respective LGSI-negative groups (Table 2). In women, but not in men, the LGSI-positives also had an increased proportion of those with diabetes. Among both men and women, medication for hypertension was more prevalent in the LGSI-positives compared to those without LGSI (Table S3).

Both men and women with LGSI more frequently self-reported CVDs and joint diseases, while more frequent reporting of pulmonary diseases was only observed in LGSI-positive women (Table 2). Among CVDs, men and women with LGSI more commonly reported coronary heart disease (angina pectoris) and/or heart failure, while more common reporting of prior myocardial infarction, stroke, and/or heart surgery was a characteristic of the LGSI-positive men, but not of the LGSI-positive women (Table S4). As for pulmonary diseases, the LGSI-positive women more frequently reported bronchial asthma, which was not observed in the LGSI-positive men. The proportions of the participants reporting neoplasms, kidney diseases, liver diseases, and the proportions of those with depression and/or anxiety did not differ between the LGSI-positive and LGSI-negative groups in men and women.

### 3.2. Socio-Demographic Characteristics

When men and women were taken together (pooled analysis) (Table 3), increased odds of LGSI were associated with older age in univariate analysis (Model 1) and after adjustments for other socio-demographic variables (sex, higher education, marital status, regular work, and occupational category), and lifestyle variables (current smoking and hazardous drinking) (Model 2). The association was attenuated to non-significance after the adjustment for cardiometabolic characteristics (Models 3–4). The same was observed in men and in women separately (Table 4). When regression analyses were performed with MS variable entered instead of its separate components (cardiometabolic conditions), age was independently associated with LGSI in the pooled sample and in men (Table 5).

Sex had no associations with LGSI after the adjustment for age (Model 1, Table 3). The same was after the adjustments for socio-demographic and lifestyle variables, but sex interacted with marital status and smoking (Model 2). When further adjusting for cardiometabolic characteristics, sex also showed an interaction with abdominal obesity, and that resulted in a reduced OR of LGSI in women compared to men (Model 3). The reduced OR in women persisted further adjustments for self-reported diseases (Model 4). In the pooled model with MS entered instead of its components, sex had interactions with marital status and smoking but no independent association with LGSI (Table 5).

The participants with higher education had reduced odds of LGSI in the pooled analysis after the adjustment for age (Model 1), but not after the following adjustments for other socio-demographic and lifestyle variables (Models 2–4, Table 3). In the sex-stratified analyses, the reduced age-adjusted odds of LGSI were observed with higher education only in women (Model 1, Table 4). The association persisted in the adjustments for other socio-demographic variables (Model 2), but ceased manifesting when cardiometabolic characteristics were entered (Models 3–4).

In the pooled analysis, odds of LGSI were reduced among married participants compared to those unmarried, but only after the adjustment for other studied socio-demographic and lifestyle variables, which was combined with the entry of the interaction term marital status by sex (*p* = 0.050) (Model 2, Table 3). The reduced odds of LGSI persisted further adjustments for cardiometabolic characteristics and self-reported diseases (Models 3–4). Corresponding to the identified interaction between marital status and sex, the reduced odds of LGSI with being married were only observed in men, not in women, and the association in men was resistant to all the adjustments (Models 1–4, Table 4). A similar performance of being married was observed when modelling LGSI with MS entered instead of its separate components (Table 5).

Being in regular paid work had no associations with LGSI in the pooled analyses (Models 1–4, Table 3). It was associated with lower odds of LGSI among men after the adjustment for age (Model 1), but not after the following adjustments for other socio-demographic and lifestyle variables (Models 2–4, Table 4).

Belonging to high-skilled and low-skilled blue-collar occupational categories was associated with increased odds of LGSI in the pooled age-adjusted model (Model 1), but not after adjustments for other socio-demographic and lifestyle variables (Models 2–4, Table 3). In the sex-stratified analyses, the increased age-adjusted odds of LGSI were found only in women in the high-skilled blue-collar category (Model 1), but not after further adjustments for socio-demographic and lifestyle variables (Models 2–4, Table 4).

### 3.3. Lifestyle Characteristics

In the pooled analyses, smokers had increased odds of LGSI compared to non-smokers irrespective of all the adjustments (Models 1–4, Table 3). In the sex-stratified analyses, the increased odds were observed in smoking men (Models 1–4), but not in smoking women (Table 4). That also manifested as an interaction between smoking and sex in the pooled analysis (*p* = 0.027) (Table 3). The associations of smoking with LGSI and its interaction with sex were similar when the analyses were performed with MS entered instead of its separate components (Table 5).

Hazardous drinking was associated with increased odds of LGSI in the pooled age-adjusted model and after adjustments for socio-demographic characteristics and smoking (Models 1–2), but not after further adjustments for cardiometabolic characteristics and self-reported diseases (Models 3–4, Table 3). In men, the odds of LGSI were increased with hazardous drinking regardless of all the adjustments (Models 1–4, Table 4). Such an association was not observed in women. Similar associations of hazardous drinking with LGSI were present when MS was entered instead of its components (Table 5).

### 3.4. Cardiometabolic Conditions

Dyslipidemia was associated with increased odds of LGSI in the pooled analyses regardless of the adjustments (Models 1, 3–4, Table 3). In sex-stratified analyses, both men and women with dyslipidemia had increased odds of LGSI after the adjustment for age (Model 1, Table 4). However, these odds were attenuated to non-significance in both sexes after the following adjustments for socio-demographic, lifestyle, and other cardiometabolic variables (Models 3–4).

Hypertension was associated with increased odds of LGSI in the pooled analyses irrespective of the adjustments (Models 1, 3–4, Table 3). In the sex-stratified analyses, the increased odds of LGSI were observed with hypertension in both men and women after the adjustment for age (Model 1, Table 4). In men, the increased odds persisted the adjustments for socio-demographic, lifestyle, and other cardiometabolic variables (Model 3), but not the adjustment for self-reported diseases (Model 4). In women, the association between hypertension and LGSI was not observed after the adjustments for socio-demographic, lifestyle, and other cardiometabolic variables (Models 3–4).

Diabetes was associated with increased odds of LGSI in the pooled analysis with the adjustment for age (Model 1), but not after the following adjustments (Table 3). The same was observed in women (Models 1, 3–4), but not in men (Table 4).

Abdominal obesity was associated with increased odds of LGSI in the pooled analyses, and the association persisted in all the adjustments (Models 1, 3–4, Table 3). The same was observed in men and in women separately (Models 1, 3–4) (Table 4). The association of abdominal obesity with LGSI was stronger in women (OR = 4.38) than in men (OR = 2.13), which was reflected in the interaction (*p* < 0.001) between abdominal obesity and sex in the pooled analysis (Table 3).

When the MS variable was included in the analyses of associations with LGSI instead of its separate components (Table 5), it showed associations with increased odds of LGSI in the pooled analyses (OR = 2.3) as well as in men (OR = 1.8) and women (OR = 2.8).

### 3.5. Self-Reported Diseases

Participants with self-reported CVDs had increased odds of LGSI in the pooled analysis after the adjustment for age (Model 1), but not after the adjustment for all other studied variables (Model 4, Table 3). Men with CVDs had increased odds of LGSI both in the age-adjusted model (Model 1) and after the adjustment for all other variables (Model 4) (Table 4). In women, the association was not observed. Similarly, when MS was entered into the model instead of its components (Table 5), self-reported CVDs were associated with increased odds of LGSI in men only.

Pulmonary diseases were associated with increased odds of LGSI in the pooled analysis, both after age-adjustment (Model 1) and after all the following adjustments (Model 4, Table 3). Women with pulmonary diseases had elevated odds of LGSI after the adjustment for age (Model 1) and after all other adjustments (Model 4, Table 4). The association of pulmonary diseases with LGSI was not observed in men. When entering MS variable instead of its components, self-reported pulmonary diseases were associated with increased odds of LGSI in the pooled model and in women, but not in men (Table 5).

Joint diseases were associated with increased odds of LGSI in the pooled analysis after the adjustment for age (Model 1), but not after the adjustment for all other studied variables (Model 4, Table 3). In the stratified analysis, joint diseases showed no association with LGSI in men (Table 4). In women, the increased odds were observed after the adjustment for age (Model 1), but not after the following adjustments (Model 4).

### 3.6. Associations between the hs-CRP Level and Cardiometabolic Biomarkers

In the pooled analyses, hs-CRP levels were higher with higher values of TC, LDL-C, TG, non-HDL cholesterol, remnant cholesterol, Apo B, and Apo B/Apo A-1 ratio, SBP and DBP, HbA1c, BMI, WC, HC, WHR, and antihypertensive and antidiabetic medication (Table 6). The hs-CRP levels were higher with lower values of HDL-C and Apo A-1.

Among the blood lipids, the strongest correlates of hs-CRP were TG (β = 0.265), remnant cholesterol (β = 0.247), and Apo B/Apo A-1 ratio (β = 0.226) (Table 6). These parameters were the leading lipid-profile correlates of hs-CRP in men and in women taken separately (Table 7). The associations of triglycerides, remnant cholesterol, and Apo B/Apo A-1 ratio in women (β = 0.335, β = 0.298, and β = 0.258, respectively) were stronger than in men (β = 0.166, β = 0.171, and β = 0.171, respectively).

DBP had a slightly stronger association with the hs-CRP level compared to SBP in the pooled analysis (β = 0.198 vs. β = 0.174) (Table 6). The difference in the associations of the two BP measurements with hs-CRP was more pronounced in men (β = 0.160 vs. β = 0.118), while in women the associations of both SBP and DBP with hs-CRP were stronger (β = 0.199 and β = 0.210, respectively) (Table 7).

In the pooled analysis, HbA1c had a modest association with hs-CRP (β = 0.149) compared to other hs-CRP correlates (Table 7), and again the association was stronger in women (β = 0.170) than in men (β = 0.114) (Table 7).

Among all the cardiometabolic biomarkers in the study, anthropometric parameters had the strongest positive associations with hs-CRP (Table 6). WHR was the leading correlate (β = 0.473), followed by WC (β = 0.460), BMI (β = 0.406), and HC (β = 0.354). In women, the associations were even stronger with WC being the lead (β = 0.530), followed by BMI (β = 0.498), HC (β = 0.400), and WHR (β = 0.400) (Table 7). In men, WHR was the leading correlate (β = 0.334), followed by WC (β = 0.292), BMI (β = 0.243), and HC (β = 0.179).

Being markers of hypertension and diabetes under treatment, self-reports of taking antihypertensives and antidiabetics also showed associations with increased hs-CRP levels in the pooled analyses (β = 0.155 and β = 0.050, respectively) (Table 6). In the sex-stratified analyses, only hypertensives showed associations with increased hs-CRP levels in both men and women (β = 0.109 and β = 0.185, respectively) (Table 7). Self-reporting the intake of lipid lowering agents (statins) showed no associations with the hs-CRP level.

## 4. Discussion

We described the distribution of LGSI in adult men and women of Arkhangelsk, Russia and identified the associated characteristics. The study contributed to the knowledge of systemic inflammation and associated factors, predictors and consequences, generated by the earlier studies in Russian populations [41,42,43,44].

Population levels of hs-CRP (the median of 1.65 mg/L in men and 1.50 mg/L in women; the geometric mean of 1.74 mg/L in men and 1.54 mg/L in women) in Arkhangelsk adult population aged 35–69 years were within the spectrum of earlier estimates for white or European populations [10,45,46]. The geometric means for both sexes were lower compared to the geometric mean of CRP for white residents in the United States (2.03 mg/L for both sexes) [45]. However, the medians for men and women were higher compared the median CRP for Europeans of both sexes in Canada (1.24 mg/l) [46], and geometric means were higher than those in the Norwegian Tromsø study (1.37 mg/L for men and 1.03 mg/L for women) [10].

### 4.1. Socio-Demographic Correlates

Our study demonstrated that hs-CRP levels increased with age, which is consistent with findings of other studies [45,47,48,49] and the inflammation theory declaring that chronic subclinical inflammation is activated with age [50].

The median level and the geometric mean of hs-CRP were higher in men, while the prevalence of LGSI had no significant difference between sexes. Previous research showed higher levels of CRP in men [51] and no sex differences [52], but the majority of studies found the higher levels in women [49,53,54,55,56,57,58]. A possible explanation of the higher hs-CRP in men in our study could be the age limitation of the studied population to 35–69 years [57]. Compared with men, women in older ages may have a higher prevalence of obesity, insulin resistance, and metabolic abnormalities, which are associated with higher levels of inflammatory markers [59,60,61,62]. Therefore, inclusion of participants older than 69 year could have balanced hs-CRP levels across sexes.

Despite no sex difference in the LGSI prevalence, sex showed interactions with marital status, smoking, and abdominal obesity in its associations with LGSI. Adjustments for these interactions resulted in reduced odds of LGSI in women, reflecting that the association between sex and LGSI depends on having the interacting factors. The sex-stratified analysis allowed describing the nature of these interactions. Based on the discovered age-adjusted associations, a man with LGSI at a screening in Arkhangelsk would most likely be of an older age, unmarried, not in regular paid work, a smoker, and a hazardous drinker. He would likely have abdominal obesity, hypertension, dyslipidemia, and a CVD in anamnesis. For comparison, woman with LGSI would likely have an older age, no higher education, no regular paid work, and would likely be a high-skilled blue-collar. She would likely have cardiometabolic conditions (abdominal obesity, hypertension, dyslipidemia, diabetes), pulmonary diseases, and joint diseases. However, when the associations with LGSI were assessed with socio-demographic variables, lifestyle variables, and cardiometabolic conditions mutually adjusted, older age, higher education, being in regular paid work, and being a blue-collar were no longer significant LGSI correlates in the pooled and sex-stratified analyses. Therefore, the associations of these socio-demographic characteristics with LGSI were likely mediated by the studied lifestyle and cardiometabolic variables. Similarly, a number of previous studies demonstrated associations of higher CRP levels with lower levels of education and income, but health behaviors and metabolic parameters explained the major part of these differences [23,63,64,65].

Being married was found associated with lower odds of LGSI in men, but not in women, and the association persisted all the adjustments. A difference in diet between married and unmarried men might be an explanation, but we did not see a difference between these groups in dietary quality. Another explanation might be a higher number of sexual partners and higher exposure to sexually transmitted infections among unmarried men, which could led to elevated hs-CRP levels. Alternatively, reduced levels of sex hormones among unmarried men could have led to lower testosterone levels, and these levels were shown to be associated with pro-inflammatory state [66]. We had no data to investigate the latter hypotheses. A better explanation requires further research.

### 4.2. Lifestyle Correlates

Smoking and hazardous drinking were more prevalent among men, and only in men, these characteristics had associations with LGSI, regardless of the adjustments. According to previous studies, the association of LGSI with smoking could be explained by the induction of local and systemic inflammation via generalized leukocytosis, platelet aggregation, and the influence on compounds of visceral adipose tissue, resulting in the release of interleukin-6 and additional CRP production in hepatic cells [25,30]. As for alcohol, our results are in line with earlier findings of the increased CRP levels with moderate and heavy drinking, which is more common in men [21,25,67].

### 4.3. Cardiometabolic Conditions

Our analyses have demonstrated LGSI associations with all four studied cardiometabolic conditions (abdominal obesity, hypertension, dyslipidemia, and diabetes), except for diabetes in men. Out of the four, abdominal obesity had the strongest association with LGSI in both men and women, being twice as strong correlate of LGSI in women compared to men. These findings suggest that LGSI in the Russian population is most commonly obesity-dependent. Such systemic inflammation was described as developing due to the accumulation of adipose tissue in the abdominal area, which activates macrophages, endothelial dysfunction, excessive production of tumor necrosis factor α, interleukin-6 and adipocytokines, oxidative stress, and insulin resistance [61,68]. The mechanism functions as the effect of fat tissue on CRP production in the liver: the increased release of IL-6 from adipose tissue occurs with increased body fat and results in elevated CRP through inducing its synthesis and secretion by hepatocytes [61]. In parallel, with obesity-related oxidative stress, different biomolecules, including lipids, can be damaged [69]. LDL and proteins (e.g., fibrinogen) are modified to oxidized particles, which provoke all stages of the atherosclerosis process, starting with endothelial dysfunction, leukocyte activation, foam cell formation, and smooth muscle cell migration and proliferation, thus resulting in platelet adhesion and aggregation [70,71]. Oxidized LDL was also shown to be associated with the incidence of MS [72,73], which reflects the complex interlinkage of the described processes in their effects on CVD development.

### 4.4. Self-Reported Diseases

We found the increased odds of LGSI with self-reported CVDs in men, but not in women. Pulmonary diseases and joint diseases were more commonly self-reported by women and were found associated with increased odds of LGSI in women, but not in men. These findings may indicate possible sex differences in organ-specific pathogenic determinants of LGSI. However, the findings are based on self-reports of diagnosed diseases, and there might be a differential bias in self-reports by men and women. Therefore, the discovered phenomenon is of interest, but its nature is not fully clear and requires further investigation using objective clinical data.

### 4.5. Cardiometabolic Biomarkers

In our study, the hs-CRP level showed associations with all studied lipid particles except for lipoprotein (a). The closest correlates of hs-CRP were TG, remnant cholesterol, and the apoB/ApoA ratio. An earlier study has shown that a high level of TG was the only one of the five MS components contributing to LGSI [74]. The recent evidence of remnant cholesterol being an increasingly sensitive marker of CVD morbidity and mortality [75,76] suggests the need for further research of its relations and possible interactions with LGSI in pathogenesis of CVDs.

The association between inflammation and metabolic phenotypes may partly be explained by a shared genetic background [17,18]. To date, there were several studies showing shared genetic polymorphisms of CRP and MS components [13,17,18,77]. Based on that, further advances in genetic research can have promising potentials to bring more light on the relation between lipid profiles and LGSI.

Previous studies have demonstrated the association of chronic inflammation with hypertension and its complications [78,79,80] and on CRP’s mediating role in the relationship between BMI and hypertension [78,79]. In our study, diastolic BP was found to be slightly stronger associated with LGSI compared to systolic BP. That may be explained by the direct involvement of CRP in the development of endothelial dysfunction, vascular stiffness, and by enhanced vascular response to angiotensin II and aldosterone [81,82]. This cascade leads to increased vascular resistance and may largely affect diastolic BP [79,80].

In additional analyses, we have shown that MS was associated with the increased odds of LGSI in both men and women. However, the associations between LGSI and MS were weaker in both sexes compared with respective associations between LGSI and abdominal obesity—an MS’s component, earlier analyzed as a separate condition. These findings indicate that abdominal obesity was the major driver of the observed MS-LGSI association.

### 4.6. Strengths and Limitations

This study has several limitations. The LGSI status was assessed using hs-CRP as the only one marker of inflammation measured at a single occasion. Thus, the participants with subacute resolving inflammation could be falsely classified as LGSI-positives, and that could have attenuated the studied associations. Blood sampling was not performed at full fasting. For this reason, the described levels of lipids, triglycerides in particular, could be partially affected by the recent food intake. The data about economic and lifestyle characteristics and diseases were collected using questionnaires. Self-reported questionnaires about lifestyle could be biased due to the desirability to undervalue socially unwelcomed attitudes [83]. The sex-stratified analyses had lower statistical power compared to the pooled analysis, which limited our ability to identify significant associations in subgroups of men and women. The participants taking anti-inflammatory medications were excluded from regression analyses, although effect of different medications on the degree of hs-CRP decrease could vary and was not evaluated. The population of younger age groups (<35 years old) was not included according to the study design, although young adults can have substantially different socio-economic and lifestyle characteristics and the inflammatory status distribution. The response rate of 68% in the KYH study could be a source of a selection bias. Because of the cross-sectional study design, the directions of the associations between LGSI and other studied parameters could not be established. Finally, the study added to the knowledge of LGSI prevalence and correlates in a Russian population with a high CVD mortality, but it may be of a limited novelty with respect to the LGSI determinants in a more general sense.

## 5. Conclusions

One-third of the adult population in Arkhangelsk had LGSI. Proportions of participants with LGSI did not differ by sex, but increased with age in both sexes. Abdominal obesity was most closely associated with LGSI both in men and in women. Apart from that, profiles of LGSI correlates were different in men and women, suggesting sex-specific features of LGSI development and health consequences.

## Figures and Tables

**Table 1:** Socio-demographic, housing, and lifestyle characteristics of men and women with and without low-grade systemic inflammation (*n* = 2054).

	Men		*p*-Value *	Women		*p*-Value *
	LGSI−	LGSI+		LGSI−	LGSI+	
*n*	546	308		752	448	
*Socio-economic characteristics*						
Age, years, M ± SD	52.8 ± 9.5	55.0 ± 9.5	0.001	52.4 ± 10.0	55.2 ± 9.4	<0.001
Higher education, Abs (%)	205 (37.6)	96 (31.2)	0.061	325 (43.2)	136 (30.4)	<0.001
Being married, Abs (%)	437 (80.0)	226 (73.4)	0.025	380 (50.5)	221 (49.3)	0.697
In regular paid work, Abs (%)	379 (69.4)	178 (57.8)	0.001	505 (67.2)	262 (58.5)	0.002
Occupation category, Abs (%)			0.141			0.024
- High-skilled white-collar (ISCO 1–3)	201 (36.8)	94 (30.5)		346 (46.0)	170 (38.0)	
- Low-skilled white-collar (ISCO 4–5)	42 (7.7)	18 (5.8)		246 (32.7)	154 (34.4)	
- High-skilled blue-collar (ISCO 6–7)	119 (21.8)	76 (24.7)		46 (6.1)	38 (8.5)	
- Low-skilled blue-collar (ISCO 8–9)	184 (33.7)	120 (39.0)		114 (15.2)	86 (19.2)	
Poor financial situation ^a^, Abs (%)	55 (10.1)	38 (12.3)	0.308	127 (16.9)	75 (16.7)	0.947
*Housing characteristics*						
Shared flat, house, or hostel, Abs (%)	13 (2.4)	13 (4.2)	0.133	28 (3.7)	22 (4.9)	0.319
No hot water amenities, Abs (%)	49 (9.0)	35 (11.4)	0.260	74 (9.8)	54 (12.1)	0.230
No central heating, Abs (%)	33 (6.0)	20 (6.5)	0.794	50 (6.7)	39 (8.7)	0.189
Household size, members, Me (Q1; Q3)	3.0 (2.0; 4.0)	3.0 (2.0; 4.0)	0.247	2.0 (2.0; 4.0)	2.0 (2.0; 3.0)	0.227
Dwelling size per member, m^2^, Me (Q1; Q3)	20.0 (14.7; 28.0)	19.8 (14.7; 27.3)	0.900	20.7 (15.0; 30.3)	21.0 (15.1; 29.8)	0.982
*Lifestyle characteristics*						
Smoking, Abs (%)	151 (27.7)	126 (40.9)	<0.001	105 (14.0)	62 (13.8)	0.690
Physical inactivity ^b^, Abs (%)	81 (14.8)	49 (15.9)	0.675	119 (15.8)	85 (19.0)	0.160
Unhealthy diet ^c^, Abs (%)	26 (4.8)	16 (5.2)	0.779	36 (4.8)	20 (4.5)	0.798
Hazardous drinking ^d^, Abs (%)	141 (25.8)	106 (34.4)	0.008	18 (2.4)	11 (2.5)	0.946

LGSI, low-grade systemic inflammation; LGSI+, LGSI-positive participants; LGSI-, LGSI-negative participants; ISCO, International Standard Classification of Occupation. ^a^ Poor financial situation was defined as self-reported difficulties to buy food or clothes; ^b^ The category includes “inactive” and “moderately inactive” participants according to the EPIC questionnaire; ^c^ Defined using dietary quality score questionnaire; ^d^ AUDIT test score ≥ 8. * Two-sample *t*-test for normally distributed continuous variables, two-sample Wilcoxon rank-sum test for variables with skewed distributions and for count variables, Pearson’s chi-squared test for categorical variables.

**Table 2:** Cardiometabolic characteristics, self-reported diseases, and mental health of men and women with and without low-grade systemic inflammation (*n* = 2054).

	Men		*p*-Value *	Women		*p*-Value *
	LGSI−	LGSI+		LGSI−	LGSI+	
*n*	546	308		752	448	
	Abs (%)			Abs (%)		
*Cardiometabolic characteristics*						
Dyslipidemia ^a^	460 (84.3)	278 (90.3)	0.014	605 (80.5)	405 (90.4)	<0.001
Hypertension ^b^	324 (59.3)	223 (72.4)	<0.001	327 (43.5)	281 (62.7)	<0.001
Diabetes ^c^	31 (5.7)	26 (8.4)	0.120	50 (6.7)	61 (13.6)	0.001
Abdominal obesity ^d^	248 (45.4)	194 (63.0)	<0.001	390 (51.9)	381 (85.0)	<0.001
*Self-reported diseases*						
Cardiovascular diseases ^e^	96 (17.6)	89 (28.9)	<0.001	158 (21.0)	132 (29.5)	0.001
Pulmonary diseases ^f^	53 (9.7)	40 (13.0)	0.140	115 (15.3)	100 (22.3)	0.002
Neoplasms ^g^	14 (2.6)	8 (2.6)	0.976	48 (6.4)	36 (8.0)	0.278
Kidney diseases ^g^	63 (11.5)	43 (14.0)	0.303	168 (22.3)	91 (20.3)	0.409
Liver diseases ^g^	80 (14.7)	42 (13.6)	0.684	139 (18.5)	81 (18.1)	0.861
Joint diseases ^h^	93 (17.0)	70 (22.7)	0.042	186 (24.7)	162 (36.2)	<0.001
*Mental health*						
Depression ^i^	118 (21.6)	75 (24.4)	0.358	275 (36.6)	161 (35.9)	0.826
Anxiety ^j^	70 (12.8)	40 (13.0)	0.944	190 (25.3)	108 (24.1)	0.653

LGSI, low-grade systemic inflammation; LGSI+, LGSI-positive participants; LGSI-, LGSI-negative participants. ^a^ Dyslipidemia was defined as total cholesterol ≥ 5.2 mmol/L, and/or triglycerides > 1.7 mmol/L, and/or LDL-cholesterol > 3.0, and/or HDL-cholesterol < 1.0 mmol/L for men, and/or HDL-cholesterol < 1.2 mmol/L for women, and/or intake of lipid lowering medication; ^b^ Hypertension was defined as systolic blood pressure (BP) > 140 mm Hg, and/or diastolic BP > 90 mm Hg, and/or self-reported intake of antihypertensives; ^c^ Diabetes was defined as glycated hemoglobin ≥ 6.5%, and/or self-reported intake of antidiabetics, and/or self-report of having been diagnosed with diabetes, followed by the statement of the diabetes type and treatment prescribed (insulin, drugs, or diet); ^d^ Abdominal obesity was defined as waist circumference > 94 cm for men, or > 80 cm for women; ^e^ Cardiovascular diseases were defined as self-reported coronary heart disease (angina pectoris), and/or myocardial infarction, and/or stroke, and/or heart failure, and/or atrial fibrillation, and/or prior heart surgery (percutaneous coronary intervention or coronary bypass); ^f^ Pulmonary diseases were defined as self-reported chronic obstructive pulmonary disease, and/or bronchial asthma, and/or chronic bronchitis; ^g^ Self-reported diseases of corresponding classes without specification; ^h^ Joint diseases were defined as self-reported osteoarthritis, and/or osteoarthritis, and/or rheumatoid arthritis; ^i^ Depression was defined as PHQ-9 score ≥ 5; ^j^ Anxiety was defined as GAD-7 score ≥ 5. * Pearson’s chi-squared test.

**Table 3:** Associations of socio-demographic, lifestyle, and cardiometabolic characteristics with low-grade systemic inflammation (*n* = 2054).

	Model 1	Model 2	Model 3	Model 4
	OR (95% CI)			
*Socio-demographic characteristics*				
Age, years	1.03 (1.02; 1.04)	1.02 (1.01; 1.04)	1.00 (0.99; 1.02)	1.00 (0.99; 1.01)
Sex, woman	1.06 (0.88; 1.27)	1.07 (0.73; 1.57)	0.59 (0.36; 0.96)	0.55 (0.33; 0.89)
Higher education	0.70 (0.57; 0.84)	0.78 (0.61; 1.01)	0.87 (0.67; 1.12)	0.88 (0.68; 1.14)
Being married	0.86 (0.71; 1.03)	0.70 (0.50; 0.98)	0.64 (0.45; 0.90)	0.62 (0.44; 0.88)
In regular paid work	0.81 (0.65; 1.00)	0.85 (0.68; 1.06)	0.83 (0.66; 1.04)	0.85 (0.68; 1.07)
Occupation category				
- High-skilled white-collar (ISCO 1–3)	*Reference*	*Reference*	*Reference*	*Reference*
- Low-skilled white-collar (ISCO 4–5)	1.27 (1.00; 1.61)	1.05 (0.79; 1.38)	1.02 (0.76; 1.36)	1.02 (0.76; 1.37)
- High-skilled blue-collar (ISCO 6–7)	1.39 (1.05; 1.84)	1.13 (0.81; 1.59)	1.14 (0.81; 1.61)	1.13 (0.80; 1.60)
- Low-skilled blue-collar (ISCO 8–9)	1.34 (1.06; 1.69)	1.09 (0.81; 1.46)	1.04 (0.77; 1.40)	1.04 (0.77; 1.41)
*Lifestyle characteristics*				
Smoking	1.44 (1.16; 1.79)	1.65 (1.22; 2.24)	1.94 (1.42; 2.66)	1.92 (1.40; 2.63)
Hazardous drinking	1.46 (1.12; 1.90)	1.42 (1.06; 1.91)	1.28 (0.95; 1.73)	1.28 (0.94; 1.73)
*Cardiometabolic characteristics*				
Dyslipidemia ^a^	1.78 (1.34; 2.37)		1.48 (1.09; 2.01)	1.46 (1.07; 1.98)
Hypertension ^b^	1.76 (1.43; 2.16)		1.28 (1.02; 1.60)	1.25 (1.00; 1.57)
Diabetes ^c^	1.61 (1.16; 2.23)		1.25 (0.89; 1.76)	1.19 (0.85; 1.69)
Abdominal obesity ^d^	3.09 (2.52; 3.78)		2.15 (1.58; 2.91)	2.13 (1.57; 2.89)
*Self-reported diseases*				
Cardiovascular diseases ^e^	1.42 (1.13; 1.78)			1.24 (0.97; 1.58)
Pulmonary diseases ^f^	1.44 (1.12; 1.84)			1.37 (1.05; 1.79)
Joint diseases ^g^	1.44 (1.17; 1.77)			1.23 (0.98; 1.54)

ISCO, International Standard Classification of Occupation. Model 1—age-adjusted for all variables except age; Model 2—all socio-economic and lifestyle variables in the table mutually adjusted with added interaction terms sex by marital status (*p* = 0.050) and sex by smoking (*p* = 0.027); Model 3—adjusted as per Model 2 plus cardiometabolic characteristics in the table and interaction term sex by abdominal obesity (*p* < 0.001); Model 4—adjusted as per Model 3 plus self-reported diseases in the table. ^a^ Dyslipidemia was defined as total cholesterol ≥ 5.2 mmol/L, and/or triglycerides > 1.7 mmol/L, and/or LDL-cholesterol > 3.0, and/or HDL-cholesterol < 1.0 mmol/L for men, and/or HDL-cholesterol < 1.2 mmol/L for women, and/or intake of lipid lowering medication; ^b^ Hypertension was defined as systolic blood pressure (BP) > 140 mm Hg, and/or diastolic BP > 90 mm Hg, and/or self-reported intake of antihypertensives; ^c^ Diabetes was defined as glycated hemoglobin ≥ 6.5%, and/or self-reported intake of antidiabetics, and/or self-report of having been diagnosed with diabetes, followed by the statement of the diabetes type and treatment prescribed (insulin, drugs, or diet); ^d^ Abdominal obesity was defined as waist circumference > 94 cm for men, or > 80 cm for women; ^e^ Cardiovascular diseases were defined as self-reported coronary heart disease (angina pectoris), and/or myocardial infarction, and/or stroke, and/or heart failure, and/or atrial fibrillation, and/or prior heart surgery (percutaneous coronary intervention or coronary bypass); ^f^ Pulmonary diseases were defined as self-reported chronic obstructive pulmonary disease, and/or bronchial asthma, and/or chronic bronchitis; ^g^ Joint diseases were defined as self-reported osteoarthritis, and/or osteoarthrosis, and/or rheumatoid arthritis.

**Table 4:** Associations of socio-demographic, lifestyle, and cardiometabolic characteristics with low-grade systemic inflammation, by sex (*n* = 2054).

	Men				Women			
	Model 1	Model 2	Model 3	Model 4	Model 1	Model 2	Model 3	Model 4
	OR (95% CI)				OR (95% CI)			
*Socio-demographic characteristics*								
Age, years	1.02 (1.01; 1.04)	1.02 (1.01; 1.04)	1.02 (1.00; 1.04)	1.01 (0.99; 1.03)	1.02 (1.01; 1.04)	1.02 (1.01; 1.04)	0.99 (0.98; 1.01)	0.99 (0.97; 1.01)
Higher education	0.78 (0.58; 1.05)	0.94 (0.63; 1.41)	0.96 (0.63; 1.46)	0.96 (0.63; 1.46)	0.64 (0.50; 0.83)	0.68 (0.49; 0.95)	0.75 (0.53; 1.06)	0.75 (0.53; 1.06)
Being married	0.63 (0.45; 0.88)	0.68 (0.48; 0.96)	0.60 (0.42; 0.85)	0.59 (0.41; 0.84)	1.01 (0.80; 1.28)	1.01 (0.80; 1.29)	1.00 (0.78; 1.29)	1.01 (0.78; 1.30)
In regular paid work	0.70 (0.50; 0.96)	0.74 (0.53; 1.03)	0.72 (0.51; 1.01)	0.75 (0.53; 1.06)	0.91 (0.68; 1.21)	0.95 (0.71; 1.27)	0.91 (0.67; 1.24)	0.93 (0.69; 1.27)
Occupation category								
- High-skilled white-collar (ISCO 1–3)	*Reference*	*Reference*	*Reference*	*Reference*	*Reference*	*Reference*	*Reference*	*Reference*
- Low-skilled white-collar (ISCO 4–5)	0.97 (0.53; 1.78)	0.87 (0.46; 1.64)	0.84 (0.44; 1.61)	0.89 (0.46; 1.72)	1.30 (0.99; 1.71)	1.05 (0.76; 1.46)	1.00 (0.71; 1.41)	0.99 (0.70; 1.40)
- High-skilled blue-collar (ISCO 6–7)	1.33 (0.91; 1.95)	1.08 (0.67; 1.72)	1.12 (0.69; 1.81)	1.12 (0.69; 1.82)	1.60 (1.00; 2.57)	1.26 (0.76; 2.10)	1.15 (0.67; 1.98)	1.12 (0.65; 1.93)
- Low-skilled blue-collar (ISCO 8–9)	1.33 (0.95; 1.87)	1.10 (0.70; 1.71)	1.05 (0.66; 1.66)	1.07 (0.67; 1.69)	1.39 (0.99; 1.95)	1.09 (0.73; 1.62)	0.99 (0.65; 1.50)	0.99 (0.65; 1.50)
*Lifestyle characteristics*								
Smoking	1.88 (1.40; 2.53)	1.68 (1.24; 2.29)	1.97 (1.43; 2.72)	1.96 (1.42; 2.70)	1.11 (0.78; 1.56)	1.02 (0.72; 1.46)	0.99 (0.68; 1.45)	0.95 (0.65; 1.39)
Hazardous drinking	1.70 (1.24; 2.33)	1.50 (1.09; 2.08)	1.45 (1.04; 2.02)	1.46 (1.04; 2.04)	1.24 (0.58; 2.67)	1.07 (0.48; 2.35)	0.75 (0.33; 1.71)	0.74 (0.33; 1.69)
*Cardiometabolic characteristics*								
Dyslipidemia ^a^	1.63 (1.05; 2.55)		1.56 (0.98; 2.50)	1.52 (0.95; 2.44)	1.90 (1.30; 2.78)		1.45 (0.97; 2.17)	1.45 (0.97; 2.18)
Hypertension ^a^	1.61 (1.17; 2.22)		1.42 (1.01; 2.00)	1.39 (0.99; 1.97)	1.95 (1.48; 2.56)		1.19 (0.88; 1.60)	1.16 (0.86; 1.57)
Diabetes ^a^	1.31 (0.75; 2.27)		1.06 (0.59; 1.91)	1.04 (0.57; 1.87)	1.79 (1.19; 2.69)		1.39 (0.91; 2.12)	1.33 (0.86; 2.04)
Abdominal obesity ^a^	2.00 (1.50; 2.67)		2.17 (1.58; 2.96)	2.13 (1.56; 2.92)	5.02 (3.70; 6.82)		4.38 (3.16; 6.06)	4.38 (3.16; 6.07)
*Self-reported diseases*								
Cardiovascular diseases ^a^	1.66 (1.16; 2.37)			1.47 (1.01; 2.15)	1.27 (0.95; 1.70)			1.06 (0.77; 1.46)
Pulmonary diseases ^a^	1.34 (0.86; 2.08)			1.23 (0.78; 1.96)	1.47 (1.09; 1.99)			1.49 (1.07; 2.07)
Joint diseases ^a^	1.32 (0.92; 1.87)			1.22 (0.83; 1.78)	1.50 (1.15; 1.96)			1.28 (0.96; 1.69)

ISCO, International Standard Classification of Occupation. Model 1—age-adjusted for all variables except age; Model 2—all socio-economic and lifestyle variables in the table mutually adjusted; Model 3—adjusted as per Model 2 plus cardiometabolic characteristics in the table; Model 4—adjusted as per Model 3 plus self-reported diseases in the table. ^a^ Definitions used are the same as in Table 3.

**Table 5:** Summarized associations of socio-economic, lifestyle, and health characteristics with low-grade systemic inflammation (*n* = 2054).

	Total Sample		Men	Women
	OR (95% CI) ^a^	*p*-Value for Interaction ^b^	OR (95% CI) ^c^	OR (95% CI) ^c^
Age, years	1.01 (1.00; 1.02)		1.02 (1.00; 1.04)	1.00 (0.99; 1.02)
Sex, woman	0.97 (0.66; 1.42)			
Being married	0.62 (0.44; 0.87)	0.017	0.63 (0.44; 0.89)	1.03 (0.80; 1.31)
Smoking	1.96 (1.44; 2.67)	0.004	1.89 (1.39; 2.58)	0.97 (0.67; 1.40)
Hazardous drinking ^d^	1.43 (1.06; 1.93)		1.55 (1.12; 2.14)	1.07 (0.47; 2.39)
Metabolic syndrome ^e^	2.32 (1.90; 2.84)		1.82 (1.33; 2.49)	2.83 (2.16; 3.70)
Cardiovascular diseases ^f^	1.22 (0.97; 1.55)		1.50 (1.03; 2.18)	1.07 (0.79; 1.46)
Pulmonary diseases ^g^	1.36 (1.05; 1.77)		1.28 (0.81; 2.02)	1.42 (1.04; 1.95)

^a^ Binary logistic linear regression with all variables in the table mutually adjusted and added interaction terms sex by marital status and sex by smoking; ^b^ Likelihood ratio test for interaction with sex. ^c^ Binary logistic regression with all variables mutually adjusted. ^d^ AUDIT test score ≥ 8; ^e^ Metabolic syndrome was defined according to the AHA/NHBLI (2009) criteria as having any three of the following five criteria: (1) waist circumference ≥ 94 cm in men and ≥80 cm in women; (2) triglycerides > 1.7 mmol/L, and/or lipid-lowering medication; (3) HDL-cholesterol < 1.0 mmol/L for men and <1.3 mmol/L for women; (4) systolic blood pressure (BP) > 130 mm Hg, and/or diastolic BP > 85 mm Hg, and/or antihypertensive medication; (5) glycated hemoglobin ≥ 5.7%, and/or antidiabetic medication; ^f^ Cardiovascular diseases were defined as self-reported coronary heart disease (angina pectoris), and/or myocardial infarction, and/or stroke, and/or heart failure, and/or atrial fibrillation, and/or prior heart surgery (percutaneous coronary intervention or coronary bypass); ^g^ Pulmonary diseases were defined as self-reported chronic obstructive pulmonary disease, and/or bronchial asthma, and/or chronic bronchitis.

**Table 6:** Associations between cardiometabolic biomarkers and low-grade systemic inflammation (*n* = 2054).

	Unadjusted Comparisons of Participants Divided by LGSI Status			Age- and Sex-Adjusted Associations with ln-Transformed hs-CRP ^a^	
	LGSI− (*n* = 1298)	LGSI+ (*n* = 756)			
	M ± SD		*p*-Value *	β	*p*-Value
*Lipid profiles*					
Total cholesterol, mmol/L	5.34 ± 1.53	5.53 ± 1.15	<0.001	0.107	<0.001
HDL-C, mmol/L	1.51 ± 0.37	1.39 ± 0.35	<0.001	−0.208	<0.001
LDL-C, mmol/L	3.58 ± 0.88	3.76 ± 0.91	<0.001	0.136	<0.001
Triglycerides, mmol/L ^b^	1.33 ± 0.97	1.70 ± 1.14	<0.001	0.265	<0.001
Non-HDL cholesterol, mmol/L	3.83 ± 1.03	4.13 ± 1.11	<0.001	0.175	<0.001
Remnant cholesterol, mmol/L	0.57 ± 0.31	0.73 ± 0.37	<0.001	0.247	<0.001
Apolipoprotein A-1 (Apo A-1), g/L	1.40 ± 0.23	1.36 ± 0.23	<0.001	−0.136	<0.001
Apolipoprotein B (Apo B), g/L	0.93 ± 0.22	1.00 ± 0.24	<0.001	0.195	<0.001
Apo B/Apo A-1 ratio	0.69 ± 0.21	0.76 ± 0.22	<0.001	0.226	<0.001
Lipoprotein(a), mg/dl ^b^	21.1 ± 27.7	22.1 ± 27.6	0.434	0.032	0.141
*Blood pressure*					
Systolic blood pressure, mm Hg	129.9 ± 19.6	134.9 ± 20.3	<0.001	0.174	<0.001
Diastolic blood pressure, mm Hg	82.1 ± 11.5	85.0 ± 11.4	<0.001	0.198	<0.001
*Blood sugar*					
HbA1c, %	5.45 ± 0.58	5.65 ± 0.88	<0.001	0.149	<0.001
*Anthropometry*					
Body Mass Index	26.1 ± 4.4	29.7 ± 5.3	<0.001	0.406	<0.001
Waist circumference, cm	86.8 ± 12.4	96.3 ± 13.0	<0.001	0.460	<0.001
Hip circumference, cm	100.4 ± 8.3	106.5 ± 10.8	<0.001	0.354	<0.001
Waist-to-hip ratio	0.86 ± 0.09	0.90 ± 0.08	<0.001	0.473	<0.001
*Medication*	Abs (%)				
Statins	125 (9.6)	81 (10.7)	0.430	−0.025	0.263
Antihypertensives	428 (33.0)	362 (47.9)	<0.001	0.155	<0.001
Antidiabetics	56 (4.3)	49 (6.5)	0.031	0.050	0.025

LGSI, low-grade systemic inflammation; LGSI+, LGSI-positive participants; LGSI-, LGSI-negative participants; β, standardized Beta coefficient; hs-CRP, high-sensitivity C-reactive protein; HDL-C, high-density lipoprotein cholesterol; LDL-C, low-density lipoprotein cholesterol; HbA1c, glycated hemoglobin. ^a^ Multiple linear regressions; ^b^ Right-skewed variables presented in the original form and analyzed in the ln-transformed form. * Two-sample *t*-test.

**Table 7:** Associations between cardiometabolic biomarkers and low-grade systemic inflammation, by sex (*n* = 2054).

	Men					Women				
	Unadjusted Comparisons of Participants Divided by LGSI Status			Age-Adjusted Associations with ln-Transformed hs-CRP ^a^		Unadjusted Comparisons of Participants Divided by LGSI Status			Age-Adjusted Associations with ln-Transformed hs-CRP ^a^	
	LGSI− (*n* = 546)	LGSI + (*n* = 308)				LGSI− (*n* = 752)	LGSI + (*n* = 448)			
	M ± SD		*p*-Value *	β	*p*-Value	Mean ± SD		*p*-Value *	β	*p*-Value
*Lipid profiles*										
Total cholesterol, mmol/L	5.26 ± 1.03	5.27 ± 1.09	0.913	0.056	0.099	5.39 ± 1.07	5.70 ± 1.19	<0.001	0.133	<0.001
HDL-C, mmol/L	1.35 ± 0.33	1.28 ± 0.34	0.003	−0.140	<0.001	1.62 ± 0.37	1.47 ± 0.34	<0.001	−0.228	<0.001
LDL-C, mmol/L	3.60 ± 0.86	3.62 ± 0.89	0.682	0.077	0.024	3.57 ± 0.90	3.86 ± 0.92	<0.001	0.169	<0.001
Triglycerides, mmol/L ^b^	1.53 ± 1.15	1.73 ± 1.13	<0.001	0.166	<0.001	1.18 ± 0.77	1.69 ± 1.15	<0.001	0.335	<0.001
Non-HDL cholesterol, mmol/L	3.91 ± 1.02	3.99 ± 1.07	0.282	0.102	0.003	3.77 ± 1.03	4.23 ± 1.12	<0.001	0.221	<0.001
Remnant cholesterol, mmol/L	0.63 ± 0.34	0.73 ± 0.36	<0.001	0.171	<0.001	0.52 ± 0.28	0.73 ± 0.38	<0.001	0.298	<0.001
Apolipoprotein A-1 (Apo A-1), g/L	1.31 ± 0.21	1.28 ± 0.22	0.016	−0.125	<0.001	1.46 ± 0.23	1.41 ± 0.22	<0.001	−0.132	<0.001
Apolipoprotein B (Apo B), g/L	0.95 ± 0.22	0.98 ± 0.23	0.070	0.130	<0.001	0.92 ± 0.22	1.02 ± 0.24	<0.001	0.234	<0.001
Apo B/Apo A-1 ratio	0.74 ± 0.21	0.79 ± 0.23	0.003	0.171	<0.001	0.64 ± 0.19	0.74 ± 0.21	<0.001	0.258	<0.001
Lipoprotein(a), mg/dl ^b^	21.5 ± 28.5	21.7 ± 27.7	0.482	0.012	0.735	20.9 ± 27.1	22.4 ± 27.6	0.077	0.045	0.115
*Blood pressure*										
Systolic blood pressure, mm Hg	136.8 ± 18.8	139.5 ± 19.2	0.048	0.118	0.001	125.0 ± 18.7	131.7 ± 20.4	<0.001	0.199	<0.001
Diastolic blood pressure, mm Hg	86.1 ± 11.2	87.9 ± 11.1	0.025	0.160	<0.001	79.3 ± 10.9	83.0 ± 11.2	<0.001	0.210	<0.001
*Blood glucose level*										
HbA1c, %	5.46 ± 0.59	5.60 ± 0.85	0.007	0.114	0.001	5.44 ± 0.57	5.68 ± 0.90	<0.001	0.170	<0.001
*Anthropometry*										
Body Mass Index	26.7 ± 4.0	28.3 ± 4.6	<0.001	0.243	<0.001	25.7 ± 4.7	30.7 ± 5.5	<0.001	0.498	<0.001
Waist circumference, cm	93.1 ± 10.6	98.5 ± 12.3	<0.001	0.292	<0.001	82.2 ± 11.5	94.9 ± 13.2	<0.001	0.530	<0.001
Hip circumference, cm	99.9 ± 7.1	102.1 ± 8.4	<0.001	0.179	<0.001	100.8 ± 9.0	109.6 ± 11.2	<0.001	0.433	<0.001
Waist-to-hip ratio	0.93 ± 0.06	0.96 ± 0.06	<0.001	0.334	<0.001	0.81 ± 0.07	0.86 ± 0.07	<0.001	0.400	<0.001
*Medication*	Abs (%)					Abs (%)				
Statins	49 (9.0)	39 (12.7)	0.089	0.019	0.585	76 (10.1)	42 (9.4)	0.681	−0.055	0.060
Antihypertensives	177 (32.4)	137 (44.5)	<0.001	0.109	0.003	251 (33.4)	225 (50.2)	<0.001	0.185	<0.001
Antidiabetics	18 (3.3)	15 (4.9)	0.252	0.040	0.251	38 (5.1)	34 (7.6)	0.074	0.052	0.071

LGSI, low-grade systemic inflammation; LGSI+, LGSI-positive participants; LGSI-, LGSI-negative participants; β, standardized Beta coefficient; hs-CRP, high-sensitivity C-reactive protein; HDL-C, high-density lipoprotein cholesterol; LDL-C, low-density lipoprotein cholesterol; HbA1c, glycated hemoglobin; ^a^ Multiple linear regressions; ^b^ Right-skewed variables presented in the original form and analyzed in the ln-transformed form. * Two-sample *t*-test.

## Data Availability

Researchers may apply for access to the Know Your Heart Study data. See data access regulations and instructions at https://metadata.knowyourheart.science (Accessed on 8 May 2023). All data requests will be guided by protecting of personal information, confidentiality agreement with participants, and their informed consents.

## Supplementary Materials

The following supporting information can be downloaded at: https://www.mdpi.com/article/10.3390/biom13050835/s1, Table S1: Summary statistics of high-sensitivity C-reactive protein levels (mg/L) in the study population by age and sex; Table S2. Proportions of the study participants with low-grade systemic inflammation by age and sex; Table S3. Medication for dyslipidemia, hypertension, and diabetes self-reported by men and women with and without low-grade systemic inflammation (*n* = 2054); Table S4. Cardiovascular and pulmonary diseases self-reported by men and women with and without low-grade systemic inflammation (*n* = 2054).
