# Supplementary material for: Socio-Demographic, Lifestyle, and Cardiometabolic Characteristics Associated with Low-Grade Systemic Inflammation in Russian Adult Population
Source: Biomolecules. 2023 May 14;13(5):835. doi: 10.3390/biom13050835 (PMC10216062; doi:10.3390/biom13050835)
Supplement: Supplementary file 1 [file biomolecules-13-00835-s001.zip › biomolecules-2281985-supplementary.pdf]

biomolecules

MDPI

Supplementary Materials

Table S1. Summary statistics of high-sensitivity C-reactive protein levels (mg/L) in the study population by age and sex

	Men					Women				
	N	M	Me	Q1	Q3	N	M	Me	Q1	Q3
Age, years										
- 35-39	92	2.80	1.33	0.84	2.65	133	2.10	0.93	0.40	2.64
- 40-44	134	3.31	1.27	0.58	2.33	207	2.34	1.07	0.49	2.97
- 45-49	134	3.05	1.10	0.61	2.86	189	2.44	1.12	0.44	2.44
- 50-54	161	3.57	1.76	0.87	3.57	188	2.90	1.51	0.66	3.54
- 55-59	151	3.66	1.68	0.88	3.65	215	3.54	1.99	0.81	3.72
- 60-64	161	4.47	1.86	1.07	3.48	202	3.69	2.16	1.15	4.96
- 65-69	154	5.84	2.24	1.13	5.10	249	4.28	1.93	0.93	4.04
*p-value for trend**			<0.001					<0.001		
Total	987	3.91	1.65	0.79	3.56	1383	3.14	1.50	0.64	3.54

* Jonckheere-Terpstra test for trend.

Table S2. Proportions of the study participants with low-grade systemic inflammation by age and sex

	Men			Women		
	N	Abs	Proportion (95% CI)	N	Abs	Proportion (95% CI)
Age, years						
- 35-39	92	28	30.4 (21.9; 40.6)	133	35	26.3 (19.5; 34.5)
- 40-44	134	32	23.9 (17.4; 31.8)	207	60	29.0 (23.2; 35.5)
- 45-49	134	41	30.6 (23.4; 38.9)	189	59	31.2 (25.0; 38.2)
- 50-54	161	56	34.8 (27.8; 42.5)	188	69	36.7 (30.1; 43.8)
- 55-59	151	48	31.8 (24.9; 39.6)	215	96	44.7 (38.1; 51.4)
- 60-64	161	63	39.1 (31.9; 46.9)	202	86	42.6 (35.9; 49.5)
- 65-69	154	63	40.9 (33.4; 48.8)	249	94	37.8 (31.9; 43.9)
*p-value for trend**			0.002			<0.001
Total	987	331	33.5 (30.7; 36.5)	1383	499	36.1 (33.6; 38.7)

* Cochran–Armitage test for trend.

Table S3. Medication for dislipidemia, hypertension, and diabetes self-reported by men and women with and without low-grade systemic inflammation (N=2054)

	Men		p-value*	Women		p-value*
	LGSI-	LGSI+		LGSI-	LGSI+	
N	546	308		752	448	
	Abs (%)			Abs (%)		
Statins	49 (9.0)	39 (12.7)	0.089	76 (10.1)	42 (9.4)	0.681
Antihypertensives	177 (32.4)	137 (44.5)	<0.001	251 (33.4)	225 (50.2)	<0.001
Antidiabetics	18 (3.3)	15 (4.9)	0.252	38 (5.1)	34 (7.6)	0.074

LGSI, low-grade systemic inflammation; LGSI+, LGSI-positive participants; LGSI-, LGSI-negative participants.* Pearson's chi-squared test.

Table S4. Cardiovascular and pulmonary diseases self-reported by men and women with and without low-grade systemic inflammation (N=2054)

	Men		p-value*	Women		p-value*
	LGSI-	LGSI+		LGSI-	LGSI+	
N	546	308		752	448	
	Abs (%)			Abs (%)		
Cardiovascular diseases						
Coronary heart disease, angina	61 (11.2)	57 (18.5)	0.003	86 (11.4)	91 (20.3)	<0.001
Myocardial infarction	28 (5.1)	36 (11.7)	<0.001	30 (4.0)	19 (4.2)	0.831
Stroke	16 (2.9)	19 (6.2)	0.022	19 (2.5)	16 (3.6)	0.298
Heart failure	35 (6.4)	40 (13.0)	0.001	80 (10.6)	68 (15.2)	0.021
Atrial fibrillation	8 (1.5)	4 (1.3)	0.843	13 (1.7)	9 (2.0)	0.726
Heart surgery [a]	19 (3.5)	24 (7.8)	0.006	2 (0.3)	3 (0.7)	0.294
Pulmonary diseases						
Chronic bronchitis or COPD	37 (6.8)	31 (10.1)	0.088	93 (12.4)	73 (16.3)	0.057
Bronchial asthma	21 (3.9)	11 (3.6)	0.839	40 (5.3)	55 (12.3)	<0.001

LGSI, low-grade systemic inflammation; LGSI+, LGSI-positive participants; LGSI-, LGSI-negative participants. [a] Percutaneous coronary intervention or coronary bypass surgery. * Pearson's chi-squared test.
